# Expression of PD-1 and Tim-3 markers of T-cell exhaustion is associated with CD4 dynamics during the course of untreated and treated HIV infection

**DOI:** 10.1371/journal.pone.0193829

**Published:** 2018-03-08

**Authors:** Norma Rallón, Marcial García, Javier García-Samaniego, Alfonso Cabello, Beatriz Álvarez, Clara Restrepo, Sara Nistal, Miguel Górgolas, José M. Benito

**Affiliations:** 1 Instituto de Investigación Sanitaria-Fundación Jiménez Díaz, Universidad Autónoma de Madrid (IIS-FJD, UAM), Madrid, Spain; 2 Hospital Universitario Rey Juan Carlos, Móstoles, Spain; 3 Hospital Universitario Carlos III-La Paz, Madrid, Spain; 4 CIBERehd., Madrid, Spain; 5 Hospital Universitario Fundación Jiménez Díaz, Madrid, Spain; University Hospital Zurich, SWITZERLAND

## Abstract

**Introduction:**

T-cell exhaustion has been involved in the pathogenesis of HIV infection. We have longitudinally analyzed PD1 and Tim3 surrogate markers of T-cells exhaustion, in parallel with other markers of HIV progression, and its potential association with CD4 changes in treated and untreated infection.

**Patients and methods:**

96 HIV patients, 49 of them followed in the absence of cART (cART-naïve group) and 47 after initiation of cART (cART group) were included and followed for a median of 43 [IQR: 31–60] months. PD1 and Tim3 expression, CD8 T-cells activation, recent thymic emigrants, activation/apoptosis and turnover of CD4 cells were assessed at baseline and during follow up. Univariate and multivariate associations with CD4 evolution were explored.

**Results:**

Parameters significantly associated with CD4 depletion in cART-naïve group were: baseline level (p = 0.02) and variation (p = 0.002) of PD1 and Tim3 co-expression on CD8, and variation of CD95 expression on CD4 (p = 0.007). Parameters significantly associated with CD4 restoration in cART group were: baseline level of CD38+HLADR- subset of CD8 (p = 0.01), variation of PD1 expression on CD8 (p = 0.036), variation of Tim3 expression on CD4 (p = 0.039) and variation of CD95 expression on CD4 (p = 0.035).

**Conclusions:**

Our results suggest that PD1 and Tim3 markers of exhaustion have a pivotal role in CD4 dynamics in HIV patients and its down-regulation would be a desirable effect of immunotherapies aimed to restore CD4 T-cell pool during progression of HIV infection.

## Introduction

Mechanisms involved in CD4 homeostasis during chronic HIV infection are not completely understood [1]. The knowledge of these mechanisms and its relative role in HIV-induced perturbation of T-cell homeostasis is relevant for the understanding of HIV immune pathogenesis and for the design of therapeutic strategies aimed to attenuate CD4 depletion in untreated infection and boost CD4 restoration after antiretroviral therapy. Alterations of different mechanisms such as T-cell activation, apoptosis, proliferation and senescence have been implicated in HIV pathogenesis [1].

More recently, T-cell exhaustion has been recognized as an important mechanism involved in T-cell dysfunction in the setting of chronic viral infections [2], including HIV [3]. Exhausted virus-specific T cells lack proliferative potential and cytokine production and this represents an important mechanism of viral persistence [4]. In chronic HIV infection, HIV-specific T cells show increased levels of exhaustion [5] and immune exhaustion correlates with HIV disease progression [6, 7]. However, T-cell exhaustion is not limited to virus-specific T cells, affecting also bulk CD4 and CD8 T-cells [3] and, in this way, perturbing overall T cell dynamics [8]. This phenomenon of T-cell exhaustion is paralleled by the expression of different surface markers that have been employed as surrogate markers of exhaustion, with PD1 and Tim3 being the ones more extensively analyzed in the setting of HIV infection [3].

The association of PD1 and Tim3 markers of exhaustion with HIV progression, as well as the association of this phenomenon with other markers involved in HIV pathogenesis, such as T-cell activation, has been previously explored in cross-sectional studies [5–7, 9, 10]. PD1 and Tim3 expression has also been explored in the context of immune restoration after cART-induced suppression of viral replication, with cross-sectional studies suggesting a role for these markers in CD4 reconstitution [11–15]. However, longitudinal studies exploring the association of PD1 and Tim3 markers with dynamics of CD4 counts in both untreated and treated HIV infection are lacking.

Based on the hypothesis that PD1 and Tim3 markers of exhaustion are involved in CD4 T-cell loss in untreated chronic HIV infection and in CD4 restoration in treated infection, the present study was designed to analyze the association of these markers with CD4 cell dynamics in the absence and in the presence of antiretroviral treatment. For this purpose, we have longitudinally analyzed the change in PD1 and Tim3 expression and other T-cell markers involved in HIV pathogenesis, and its potential association with the change in CD4 counts, in two different groups of chronically HIV-infected patients with a long-term follow up period: one naïve for cART (cART naïve group) and one treated with cART (cART group).

## Patients and methods

### Study population

This is a retrospective longitudinal study including two different groups of patients with chronic HIV infection: one with a long-term period of follow up in the absence of any antiretroviral therapy (cART naïve group), and another with a long-term period of follow up from the initiation of cART and maintaining undetectable levels of plasma HIV-RNA (cART group). Inclusion criteria were: adult with chronic HIV infection and regular immunovirological (CD4 counts and plasma HIV-RNA load) follow up, naïve for cART during the follow up period (for the cART naïve group), initiating cART and achieving and maintaining undetectable plasma HIV-load during the follow up period (for the cART group), naïve for anti-HCV treatment (for those patients co-infected with HCV), and with a cellular sample at the beginning of follow up (baseline sample) available for immunophenotyping. Sample size was calculated assuming a statistical power of 80% and Pearson correlation coefficients of 0.4 or higher in the cross-sectional analyzes. For this a sample size of 45 subjects was necessary. A total of 96 chronically HIV-infected patients were included: 49 belonging to cART naïve group (17 of them coinfected with HCV) and 47 belonging to cART group (19 of them coinfected with HCV). Twenty age and sex-matched healthy individuals were included as control group (HC). There was no overlapping between the two groups of patients (meaning that no patient was included in the two groups). To participate in the study, written informed consent was obtained from all individuals, and the study protocol was evaluated and approved by the Ethical Committee of Hospital Carlos III-La Paz.

### Cell samples

All analyzes were done in cryopreserved peripheral blood mononuclear cells (PBMCs), isolated as previously described [16]. Viability of thawed PBMCs was checked using trypan blue dye exclusion and was always greater than 85%. A baseline sample of PBMCs (at the beginning of follow up) was available for all patients included in the study. In addition, a second PBMC sample after a long period of follow up (long-term follow up sample; LT sample) was available for 26 patients (14 from patients in the cART naïve group and 12 from patients in the cART group). For patients included in the cART group, the period between baseline samples and initiation of cART was no longer than 6 months.

### Immunophenotypic study

We evaluated several CD4 and CD8 T cell parameters associated to HIV pathogenesis (including surrogate markers of: differentiation, senescence, activation, recent thymic emigrants [RTE], exhaustion, proliferation, and apoptosis) by multiparameter flow cytometry as previously described [16]. The different monoclonal antibodies and fluorochromes as well as staining conditions used in the study are shown in **S1 Table** and **S1 Text**, respectively. Moreover, **S1 Fig** shows a representative flow cytometry experiment and the strategy of gating used to analyze the different subsets of CD4 and CD8 cells.

### Statistical analysis

Different characteristics of the different groups of subjects and immune parameters analyzed are given as median [interquartile range]. Non-parametric Kruskall-Wallis test (for multiple comparisons) and Mann-Whitney U-test (for two groups comparisons) was used to test differences between groups. Associations were explored using Spearman´s rho or Pearson correlation coefficients. Linear regression analyzes were performed to ascertain which immune parameters were significantly associated with change in CD4 counts. All analyzes were done with SPSS software v15 (SPSS Inc., Chicago, IL, USA) and statistical significance was considered only when two-tailed p-values were lower than 0.05.

## Results

### Characteristics of the study population

**Table 1** shows different characteristics of patients at baseline. There were no significant differences between patient´s groups in age, gender, length of follow up, prevalence of HCV coinfection and risk group for HIV infection. In cART naïve group compared to cART group, baseline CD4 counts were significantly higher and baseline plasma HIV load significantly lower.

**Table 1:** Characteristics of patients included in the study.

Characteristic	cART naïve group (n = 49)	cART group (n = 47)	p-value
Median age (years)^1^	47 [43–51]	46 [40–51]	0.84
Gender (% of males)	84%	85%	0.85
CD4 count at baseline (cells/μl)	675 [499–799]	312 [273–378]	**<0.0001**
CD4 count at end of follow up (cells/μl)	344 [270–473]	624 [495–820]	**<0.0001**
HIV-RNA at baseline (log copies/mL)	3.8 [3.2–4.5]	4.6 [4.2–4.9]	**<0.0001**
HIV-RNA at end of follow up (log copies/mL)	4.5 [3.9–4.8]	1.7 [–]	**<0.0001**
Length of follow up (months)	43 [27–57]	42 [32–60]	0.54
Patients with HCV coinfection (%)	17 (35%)	19 (40%)	0.56
HCV-RNA in HCV+ patients (log copies/mL)	5.8 [5.2–6.3]	6.2 [4.9–6.8]	0.98
Group risk for HIV infection			0.73
Intravenous drug user (IDU)	30%	30%	
Heterosexual	15%	21%	
Male sex with male (MSM)	55%	49%	
cART regimen			
PI-based	-	38%	-
NNRTI-based	-	36%	-
NRTI-based	-	16%	-
II-based	-	10%	-

^1^ Data for continuous variables are given as median [interquartile range; IQR]

PI: protease inhibitors; NNRTI: non-nucleoside retrotranscriptase inhibitors; NRTI: nucleoside retrotranscriptase inhibitors; II: integrase inhibitors

### Change in CD4 counts during the follow up period in cART naïve and cART groups of patients

Change in CD4 counts was monitored in cART naïve and in cART groups for a median of 43(27–57) and 42(32–60) months respectively. In the cART naïve group, CD4 counts significantly decreased during follow up (from 675 (499–799) to 344 (270–473) cells/μL, p<0.0001, **Table 1**) with a median decrease of 220 (123–406) cells/μL. A similar change in CD4 was observed in HIV and in HIV/HCV patients. CD4 slope was calculated for each patient using a linear regression analysis. Of the 49 patients, 18 presented a significant decline of CD4 counts and the remaining 31 did not. All parameters were similar between the two groups of patients, with the exception of CD4 at the end of follow up, delta CD4 (ΔCD4, defined as the total variation of CD4 counts between the end and the beginning of follow up) and slope of CD4 (**S2 Table**).

In the cART group, CD4 counts significantly increased during follow up (from 312 (273–378) to 624 (495–820) cells/μL, p<0.0001, **Table 1**), with a median increase of 336 (210–491) cells/μL and no differences between HIV and in HIV/HCV patients. Of the 47 patients, 34 presented a significant increase of CD4 counts and 13 did not. There were no significant differences between these two groups of patients, except for the CD4 slope, the ΔCD4 and the CD4 counts at the end of follow up (**S2 Table**).

### Baseline levels of immune parameters in the whole population of patients

At baseline, several CD4 and CD8 T cell subsets were significantly altered in HIV patients with respect to healthy controls. Overall, alterations were more pronounced in cART group than in naïve group, likely as a consequence of the lower baseline CD4 counts observed in the cART compared to the naïve group and some of these alterations with respect to healthy controls were significant only for the cART group of patients (**S2 and S3 Figs**). There was a significant increase of Ki67 expression, especially in the CD31- (non RTE) subset of CD4 cells. Also, CD95 expression was increased in the whole population of CD4 cells and in several subsets, especially in CD31- and in Ki67+ subsets. PD1 and Tim3 markers of exhaustion were also increased in bulk CD4 cells and in several subsets of CD4 cells, with the highest levels observed in CD4+CD45RA- subset (**S2 Fig**).

Regarding CD8 T-cells subsets, HIV patients presented a significant increase in PD1 and Tim3 markers and in activation levels compared to healthy controls (**S3 Fig**), and interestingly the profile of expression of PD1 and Tim3 markers varied according to the activation status of CD8 T-cells. In resting CD8 T-cells only the expression of Tim3 was increased, whereas in activated subsets (CD38+ and/or HLADR+ subsets), both PD1 and Tim3 markers were increased (**S3 Fig**).

### Changes in immune parameters during the follow up period in the absence of therapy

The change in immune parameters after long-term follow up (LT) (median follow up 41 (27–53) months) was evaluated in the 14 patients that have both the baseline and the LT samples for analysis. This subgroup of patients employed for the longitudinal analysis was representative of the whole cART naïve group regarding the characteristics shown in **Table 1.**

**Fig 1:**
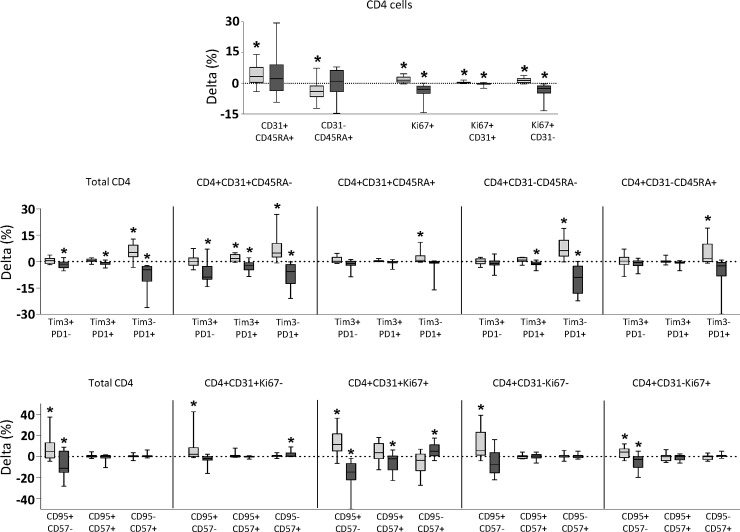
Delta values of different subsets of CD4 T-cells after long-term follow up, in cART naïve (boxes in light grey) and in cART (boxes in dark grey) groups of patients. Delta values of subsets defined by CD45RA, KI67 and CD31 markers are shown in the upper row graph. Delta values of PD1 and Tim3 expression in subsets defined according to CD45RA and CD31 markers are shown in the middle row graph. Delta values of CD95 and CD57 expression on subsets defined according to CD31 and Ki67 markers are shown in lower row graph. Delta values significantly different from zero are marked with an asterisk.

Several T-cell subsets experienced a significant variation after long term follow up (ΔLT). Among naïve (CD45RA+) CD4 cells, those expressing CD31 significantly increased. The level of proliferating (Ki67+) CD4 cells did significantly increase in both CD31+ and CD31- subsets (**Fig 1**). Markers of exhaustion (mainly PD1) increased in bulk CD4 cells and in all subsets according to CD45RA and CD31 markers. Expression of CD95 also increased in bulk CD4 cells and in many of the subsets defined by CD31 and Ki67 (**Fig 1**).

**Fig 2:**
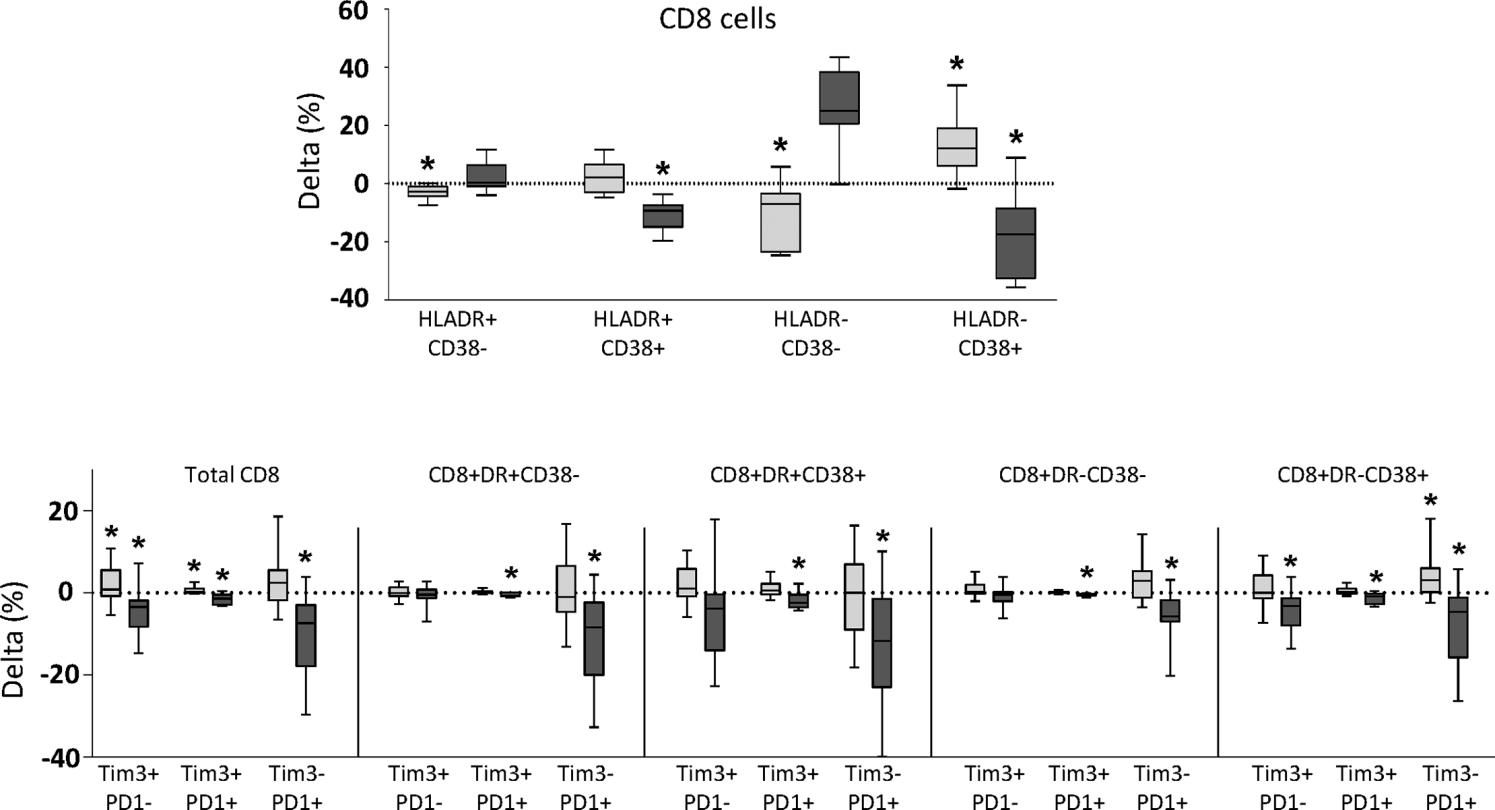
Delta values of different subsets of CD8 T-cells after long-term follow up, in cART naïve (boxes in light grey) and in cART (boxes in dark grey) groups of patients. Delta values of subsets defined by HLADR and CD38 markers are shown in the upper row graph. Delta values of PD1 and Tim3 expression in subsets defined according to HLADR and CD38 markers are shown in the lower row graph. Delta values significantly different from zero are marked with an asterisk.

With respect to CD8 subsets, HLADR-CD38+ subset significantly increased, whereas non-activated (HLADR-CD38-) cells significantly decreased (**Fig 2**). PD1 and Tim3 exhaustion markers significantly increased in total CD8 cells and in HLADR-CD38+ CD8 cells.

### Immune parameters associated with change in CD4 counts in the absence of therapy

The potential association between immune parameters and the change in CD4 counts during follow up was explored. For each patient, CD4 variation was expressed as the slope of CD4 (calculated by linear regression analysis) and as the ΔCD4 (the difference between CD4 counts at the end and at the beginning of follow up). Associations were explored with both the baseline values of the immune parameters and with the variation of the parameters after long term follow up (ΔLT).

In the bivariate analysis, significant associations were found between CD4 slope and baseline values of exhaustion of different subsets of CD8 T-cells (**S3 Table**). These associations were independent of baseline CD4 counts, baseline plasma HIV-RNA and HCV status. In a multivariate regression analysis including these CD8 subsets as explanatory variables, exhaustion (Tim3+PD1+) of CD38-HLADR- subset of CD8 cells was the only parameter significantly associated with CD4 slope (**S3 Table**).

**Fig 3:**
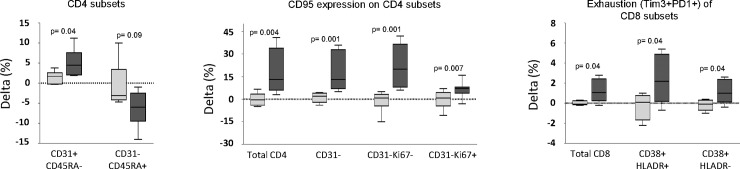
Box-plot graphs showing the delta values of different CD4 and CD8 T-cell subsets after long-term follow up in the cART naïve group of HIV patients. Patients were stratified according to the significance of their individual CD4 slope during follow up into two groups: those without (light grey boxes) and with (dark grey boxes) a significant CD4 slope. p-values for the comparison between groups (Mann-Whitney test) are given.

We found several significant associations between change in CD4 counts (ΔCD4) and variation of immune parameters during follow up (ΔLT) in the bivariate analysis. Inverse associations were found with ΔLT of CD95+ subset of CD4 cells, ΔLT of CD31+CD4RA- subset of CD4 cells, and with ΔLT of exhaustion of CD8 cells; whereas direct association was found with ΔLT of CD31-CD45RA+ subset of CD4 cells (**S4 Table**). Scatter-plots graphs of the most relevant immune parameters correlated with variation of CD4 counts are shown in **S4 Fig**. Overall, the existence of these correlations further corroborates the changes observed in the different immune parameters as described in the section above. In agreement with these associations, ΔLT of these immune parameters were significantly different in patients who experienced a significant decline of CD4 during follow up compared with those who did not (**Fig 3**).

### Evolution of immune parameters during the follow up period after initiation of cART

The change in immune parameters after long-term (LT) follow up (median follow up 34 (26–47) months) was evaluated in the 12 patients that have both the baseline and the LT samples for analysis. This subgroup of patients employed for the longitudinal analysis was representative of the whole cART group regarding the characteristics shown in **Table 1.**

Several T-cell subsets experienced a significant variation after long term follow up (ΔLT). Levels of proliferating (Ki67+) CD4 cells did significantly decrease in both CD31+ and CD31- subsets (**Fig 1**). PD1 and Tim3 markers of exhaustion significantly decreased in total CD4 cells and in the majority of subsets defined by CD45RA and CD31. Expression of CD95 decreased in total CD4 cells, especially in proliferating (ki67+) subsets (**Fig 1**).

Regarding CD8 T-cells, activation (HLADR-CD38+ and HLADR+CD38+ subsets) significantly decreased, whereas the proportion of non-activated (HLADR-CD38-) cells significantly increased (**Fig 2**). PD1 and Tim3 markers of exhaustion significantly decreased in total CD8 T-cells and in activated and resting subsets of CD8 T-cells (**Fig 2**).

### Immune parameters associated with change in CD4 counts after initiation of cART

Associations between change in CD4 counts and immune parameters were explored with both the baseline values of the immune parameters and with the variation of the parameters after long term follow up (ΔLT).

In the bivariate analysis, baseline values of HLADR-CD38+ subset of CD8 cells, co-expression of PD1 and Tim3 on HLADR+CD38- CD8 cells, and Tim3 expression on CD45RA-CD31+ CD4 cells were significantly associated with ΔCD4. In a multivariate linear regression analysis, the only baseline immune parameter associated with ΔCD4 was HLADR-CD38+ subset of CD8 cells, after correcting for CD4 counts, baseline plasma HIV-RNA HCV coinfection, age and sex (**S5 Table**). Thus, patients showing higher levels of CD8+HLADR-CD38+ cells were those showing higher increases of CD4 counts after cART.

**Fig 4:**
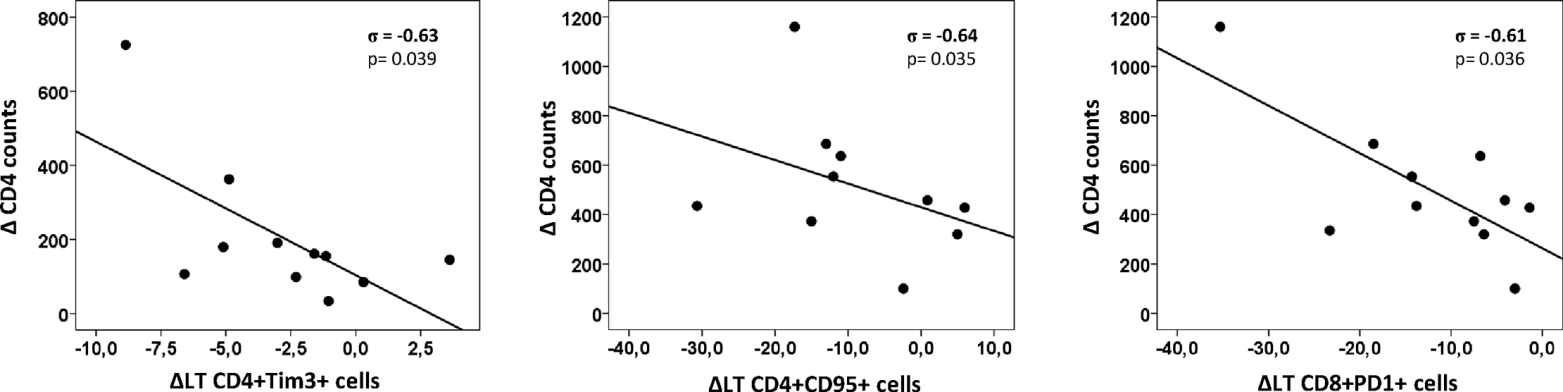
Scatter-plot graphs showing the association between variation of CD4 counts (Δ CD4 counts) and variation of immune parameters after long-term follow up (ΔLT) in patients on cART. Spearman Rho (σ) coefficient and p-value are shown in each scatter plot.

We also found several associations between CD4 evolution and variation of immune parameters during follow up (ΔLT) in the bivariate analysis (**S6 Table**). Inverse associations were found between ΔCD4 and ΔLT of: Tim3 expression on total CD4 cells and on CD45RA+CD31- subset of CD4 cells, Tim3+PD1- expression on CD45RA-CD31- subset of CD4 cells, CD95 expression on CD4 cells, PD1 expression on total CD8 cells, and on HLADR-CD38- and HLADR-CD38+ subsets of CD8 cells. Thus, patients showing higher reductions in PD1 and/or Tim3 expression on CD4 and CD8 cells and/or higher reductions in CD95 expression on CD4 cells were those showing higher increases of CD4 counts after cART (**Fig 4**).

## Discussion

The present study was designed to analyze the role of PD1 and Tim3 markers of exhaustion in CD4 dynamics in HIV-infected patients in two different situations: patients not receiving cART and showing decline of CD4 counts, and patients on suppressive cART and showing variable levels of CD4 restoration. Our results show a significant impact of PD1 and Tim3 expression on CD4 variation in both scenarios, highlighting the role of these markers in HIV disease. Moreover, the impact of PD1 and Tim3 expression on CD4 dynamics was independent of other markers of HIV pathogenesis already known for its association with CD4 counts, such as CD8 activation [17–19] or CD4 apoptosis [20, 21].

We observed that, at baseline, HIV patients included in our study showed important variations in several CD4 and CD8 T-cell subsets compared to healthy subjects. CD95 and Ki67 expression were increased as has been already reported [22, 23]. Of note, CD95 expression was increased in both Ki67+ and Ki67- subsets of CD4 T-cells. CD95 is an apoptotic marker and has an important role in HIV pathogenesis [24]. However, it is also up-regulated after activation of T-cells [25] and thus the increased levels of CD95 we found on CD4+Ki67+ cells could be linked to the activation status of this subset of CD4 T-cells and not necessarily indicative of apoptosis.

Interestingly, high levels of expression of PD1 and Tim3 markers of exhaustion appeared to be a reliable indicator of HIV disease pathogenesis. In CD8 and CD4 T-cells high levels of PD1 and/or Tim3 have been previously described [7–10, 26, 27]. In our results, CD4 T-cells showed an increased expression of both Tim3 and PD1 markers in agreement with a previous report [26] and the level of expression of these markers was different depending on the CD4 subset considered, being highest in CD45RA- subset and lowest in CD45RA+CD31+ subset. Overall, levels of expression of PD1 were higher compared to Tim3, what suggests different associations with functional impairment of CD4 T-cells for PD1 and Tim3 [28], as well as different regulatory mechanisms [29]. Simultaneous expression of PD1 and Tim3 was very infrequent and probably associated with a more exhausted phenotype with a diminished ability to secrete cytokines in HIV patients, as has been previously shown [26].

As with previous reports [7–10, 26, 27], expression of PD1 and Tim3 was also increased in CD8 T-cells, with differential expression of these markers in different CD8 subsets defined by CD38 and HLADR markers. Surprisingly, our results show that PD1 and Tim3 were increased not only in activated but also in resting CD8 T-cells, suggesting that mechanisms other than activation [10, 29] are regulating the level of expression of these markers on CD8 T-cells in HIV infection.

We examined the association of immune parameters with the variation of CD4 counts in untreated patients, and found a principal role for PD1 and Tim3 markers of exhaustion. Interestingly, level of co-expression of PD1 and Tim3 on CD38-HLADR- subset of CD8 cells at baseline was the only parameter significantly associated with the level of CD4. In agreement with this, a recent study in patients with primary HIV infection has shown that co-expression of PD1 and Tim3 on CD8 cells is directly correlated with the rate of CD4 decline during follow up [30]. However the association was stronger for CD8 cells expressing CD38 what is in contrast with our findings showing an association of CD4 decline with co-expression of PD1 and Tim3 on the subset of CD8 cells lacking both CD38 and HLADR expression. This suggests that the mechanisms underlying the association between exhaustion and CD4 dynamics may be different in acute and chronic infection.

Both PD1 and Tim3 markers have been associated with HIV disease progression in cross-sectional studies [5–7, 9] and their co-expression on T cells has been linked to a more exhausted phenotype in different chronic viral infections [31] and in HIV infection [26]. Since exhaustion and activation of T cells are linked [10, 29], co-expression of these markers on a subset of CD8 cells not expressing activation markers may be indicative of a more dysfunctional phenotype [9], likely involved in deregulation of CD4 T cell homeostasis as the results of our study suggest. Levels of this CD8 subset did not increase over time in untreated patients, suggesting that this subset is established early after infection and remains stable, in agreement with the results of a recent study [26]. In contrast, co-expression of PD1 and Tim3 on total CD8 cells and on activated subsets of CD8 cells did increase over time in untreated patients, and this increase was associated with CD4 count decline. Since expression of PD1 and Tim3 in CD8 cells has been associated with impaired proliferation [5, 6], low cytokine production [9] and cell survival [32], the rising levels of these markers we observed most likely will diminish the ability of CD8 cells to control viral replication, accelerating CD4 depletion. In fact we observed higher levels of plasma HIV-RNA at the end of follow up in untreated patients.

Our findings also support an important role for PD1 and Tim3 markers of exhaustion in CD4 restoration after cART. In agreement with previous studies [5, 6, 7, 9], we found that expression of PD1 and Tim3 on CD4 and CD8 T cells significantly decreased after cART initiation. This reduction was significantly associated with the level of CD4 restoration, meaning that higher reductions in the level of expression of PD1 and/or Tim3 were paralleled with higher increases in CD4 counts. Only two previous cross-sectional studies have analyzed the expression of PD1 in patients with different degrees of CD4 restoration after cART, and have found increased levels of PD1 on T cells in patients with poor restoration [14, 15]. Our results extend these observations using a longitudinal design and showing that change in PD1 and/or Tim3 expression and change in CD4 counts are associated, supporting an important role for these markers in CD4 homeostasis after cART. Lowering expression of PD1 and/or Tim3 markers on CD8 T cells may indirectly impact on CD4 reconstitution by restoring their ability to better control viral replication [5, 6]. On the other hand, decreasing expression of these markers of exhaustion on CD4 cells may increase their proliferative capacity [11], contributing to replenish the CD4 pool in combination with decreasing levels of CD4 apoptosis that we also found to be associated with the extent of CD4 restoration.

Finally, there are some caveats in our study that need to be mentioned: Sample size for the longitudinal analyzes was low due to the lack of cellular samples at the end of follow up in of patients. However, in spite of the low sample size, there were no differences in baseline characteristics between patients employed for the longitudinal analysis and the whole group of studied patients in both cART naïve and cART groups, and thus there was not a selection bias, being these subgroups of patients representative of the whole population of patients included in the study. Nonetheless, the low sample size used in the longitudinal analysis precluded us to perform multivariate analysis to control for other potential factors involved in CD4 count variation and thus further studies in larger cohorts are needed to elucidate the actual contribution of other important variables implied in the CD4 dynamics.

In summary, the results of our study demonstrate a significant association of PD1 and Tim3 markers of exhaustion with CD4 dynamics both in untreated and treated HIV infection, highlighting the important role of these markers in HIV pathogenesis. From a clinical point of view, our results prompt the necessity of further studies in large populations of treated patients to ascertain the implication of PD1 and Tim3 markers in failing CD4 restoration and, in that case, the design of therapeutic strategies aimed to attenuate T cell exhaustion in order to improve CD4 restoration.

## Supporting information

S1 Text Staining protocol for immunophenotypic analysis. (DOC)

S1 Table Monoclonal antibodies and fluorochromes used in the different staining panels. (DOC)

S2 Table Characteristics of cART naïve and cART groups according to significance of CD4 slope during follow up. (DOC)

S3 Table Bivariate and multivariate analysis of baseline levels of immune parameters associated with CD4 slope during follow up in the absence of therapy. (DOC)

S4 Table Bivariate analysis of associations between delta of immune parameters after LT (ΔLT) and delta CD4 during follow up in the absence of therapy. (DOC)

S5 Table Bivariate and multivariate analysis of baseline levels of immune parameters associated with variation of CD4 count (ΔCD4) during follow up after initiation of cART. (DOC)

S6 Table Bivariate analysis of associations between delta of immune parameters after LT (ΔLT) and delta CD4 (ΔCD4) during follow up after initiation of cART. (DOC)

S1 Fig Representative flow cytometry example showing the gating strategy employed to analyze the different subsets of T cells in staining panels 1, 2 and 3. Numbers inside the dot-plots represents percentages of cells in each quadrant of the plot.
(TIF)

S2 Fig Box-plot graphs showing the levels of CD4 T-cells subsets at baseline in healthy controls (white boxes), in the cART naïve (light grey boxes) and in the cART (dark grey boxes) group of patients. Upper graphs show levels of different CD4 subsets on the basis of CD45RA, CD31 and Ki67 expression. Middle graph shows levels of PD1 and Tim3 markers on different subsets of CD4 cells defined by CD31 and CD45RA markers; and lower graph the levels of activation/apoptosis (CD95) and senescence (CD57) on different subsets of CD4 cells defined by CD31 and Ki67 markers. Statistically significant differences between the three groups (by Kruskall-Wallis test) are marked by an asterisk, significant differences between cART naïve and cART groups of patients (by Mann-Whitney U test) are marked by ¶ symbol, and significant differences of each patient´s groups with respect to healthy controls (by Mann-Whitney U test) are marked by # symbol.
(PPT)

S3 Fig Box-plot graphs showing the levels of CD8 T-cells subsets at baseline in healthy controls (white boxes), in the cART naïve (light grey boxes) and in the cART (dark grey boxes) group of patients. Upper graph shows levels of different CD8 subsets on the basis of expression of activation markers CD38 and HLADR. Lower graph shows levels of PD1 and Tim3 markers on different subsets of CD8 cells defined by the expression of activation markers CD38 and HLA DR. Statistically significant differences between the three groups (by Kruskall-Wallis test) are marked by an asterisk, significant differences between cART naïve and cART groups of patients (by Mann-Whitney U test) are marked by ¶ symbol, and significant differences of each patient´s groups with respect to healthy controls (by Mann-Whitney U test) are marked by # symbol.
(PPT)

S4 Fig Scatter-plot graphs showing the association between variation of CD4 counts (CD4 slope or delta CD4) and baseline levels or variation of immune parameters after long-term follow up (ΔLT) in patients off cART. Pearson r or Spearman Rho (σ) coefficients and p-value are shown in each scatter plot.
(PPT)
