# Supplementary material for: Expression of PD-1 and Tim-3 markers of T-cell exhaustion is associated with CD4 dynamics during the course of untreated and treated HIV infection
Source: PLoS One. 2018 Mar 8;13(3):e0193829. doi: 10.1371/journal.pone.0193829 (PMC5843247; doi:10.1371/journal.pone.0193829)
Supplement: S1 Table — (DOC) [file pone.0193829.s002.doc]

**S1 Table**: Monoclonal antibodies and fluorochromes used in the different staining panels.

| **Antibody** | **Panel #1** | **Panel #2** | **Panel #3** | **Fluorochrome** | **Clone** | **Provider** |
| --- | --- | --- | --- | --- | --- | --- |
| CD4 | X |  | X | PerCP-Vio700 | VIT4 | Miltenyi Biotec |
| CD8 |  | X |  | ECD | Thy2D3 | Beckman Coulter |
| CD31 | X |  | X | FITC | WM59 | BD Biosciences |
| CD38 |  | X |  | FITC | T16 | Beckman Coulter |
| Tim3 | X | X |  | PE | 344823 | R&D Systems |
| Ki67 |  |  | X | PE | 20Raj1 | eBioscience |
| CD45RA | X |  |  | ECD | 2H4 | Beckman Coulter |
| CD57 |  |  | X | PE-CF594 | NH-1 | BD Biosciences |
| HLADR |  | X |  | PE-Cy5 | Immu357 | Beckman Coulter |
| CD95 |  |  | X | PE-Cy7 | DX2 | BD Biosciences |
| PD1/biotin | X | X |  | Streptavidin-PECy7 | J105 | eBioscience |
