# Supplementary material for: Expression of PD-1 and Tim-3 markers of T-cell exhaustion is associated with CD4 dynamics during the course of untreated and treated HIV infection
Source: PLoS One. 2018 Mar 8;13(3):e0193829. doi: 10.1371/journal.pone.0193829 (PMC5843247; doi:10.1371/journal.pone.0193829)
Supplement: S2 Table — (DOC) [file pone.0193829.s003.doc]

**S2 Table**. Characteristics of cART naïve and cART groups according to significance of CD4 slope during follow up.

|  | **cART naïve group (n=49)** | | | |  | **cART group (n=47)** | | | |
| --- | --- | --- | --- | --- | --- | --- | --- | --- | --- |
| **Characteristic** | **All** | **Non significant CD4 slope (n=31)** | **Significant CD4 slope (n=18)** | **p-value^1^** |  | **All** | **Non significant CD4 slope (n=13)** | **Significant CD4 slope (n=34)** | **p-value^1^** |
|  |  |  |  |  |  |  |  |  |  |
| Age (years) | 47 [43, 51] | 46 [42, 49] | 48 [45, 52] | 0.29 |  | 46 [40, 51] | 47 [40, 52] | 46 [38, 51] | 0.58 |
|  |  |  |  |  |  |  |  |  |  |
| Gender (% of males) | 84% | 77% | 94% | 0.12 |  | 85% | 85% | 85% | 0.64 |
|  |  |  |  |  |  |  |  |  |  |
| CD4 at beginning of follow up (cells/µL) | 675 [499, 799] | 555 [468, 703] | 761 [659, 913] | **0.004** |  | 312 [273, 378] | 318 [269, 403] | 311 [165, 38] | 0.64 |
|  |  |  |  |  |  |  |  |  |  |
| CD4 at end of follow up (cells/µL) | 344 [270, 473] | 366 [281, 590] | 331 [238, 421] | 0.28 |  | 624 [495, 820] | 516 [402, 641] | 700 [526, 853] | **0.02** |
|  |  |  |  |  |  |  |  |  |  |
| Delta CD4 (cells/µL) | -220 [-406, -123] | -166 [-244, -98] | -439 [-603, -248] | **<0.0001** |  | 336 [210, 491] | 138 [77, 370] | 379 [273, 557] | **0.006** |
|  |  |  |  |  |  |  |  |  |  |
| CD4 slope (cells/µL/month) | -4.1 [-10, -2] | -3.1 [-4.8, -1.2] | -9.2 [-15, -5.3] | **<0.0001** |  | 5.9 [3.4, 11] | 3.1 [0.9, 4.9] | 8.0 [4.5, 12] | **0.001** |
|  |  |  |  |  |  |  |  |  |  |
| HIV-RNA at beginning of follow up (log copies/mL) | 3.8 [3.2, 4.5] | 3.7 3.3, 4.3] | 4.0 [3.1, 4.3] | 0.74 |  | 4.6 [4.2, 4.9] | 4.6 [4.0, 4.9] | 4.6 [4.2, 4.9] | 0.69 |
|  |  |  |  |  |  |  |  |  |  |
| HIV-RNA at end of follow up (log copies/mL) | 4.5 [3.9, 4.8] | 4.4 [3.8, 4.9] | 4.6 [3.9, 4.7] | 0.91 |  | 1.7 | 1.7 | 1.7 | - |
|  |  |  |  |  |  |  |  |  |  |
| HCV coinfection (%) | 35% | 39% | 28% | 0.44 |  | 40% | 31% | 44% | 0.40 |
|  |  |  |  |  |  |  |  |  |  |
| Group risk for HIV infection (%) |  |  |  | 0.15 |  |  |  |  | 0.58 |
| Intravenous drug user (IDU) | 30% | 34% | 22% |  |  | 30% | 23% | 33% |  |
| Heterosexual | 15% | 21% | 6% |  |  | 21% | 23% | 20% |  |
| Males sex with male (MSM) | 55% | 45% | 72% |  |  | 49% | 54% | 47% |  |

(1): p-value for comparison between groups according to significance of CD4 slope
