# Supplementary material for: Expression of PD-1 and Tim-3 markers of T-cell exhaustion is associated with CD4 dynamics during the course of untreated and treated HIV infection
Source: PLoS One. 2018 Mar 8;13(3):e0193829. doi: 10.1371/journal.pone.0193829 (PMC5843247; doi:10.1371/journal.pone.0193829)
Supplement: S3 Table — (DOC) [file pone.0193829.s004.doc]

**S3 Table**. Bivariate and multivariate analysis of baseline levels of immune parameters associated with CD4 slope during follow up in the absence of therapy.

|  | **CD4 slope** | | | | |
| --- | --- | --- | --- | --- | --- |
|  |  | | | | |
|  | Bivariate analysis |  | Multivariate linear regression analysis | | |
|  |  |  |  |  |  |
| **Baseline values of exhaustion of different CD8 subsets** | **Pearson coefficient (p-value)** |  | **R of the model** | **Regression coefficient (ß±SE)** | **p-value** |
|  |  |  |  |  |  |
|  |  |  |  |  |  |
| Tim3+PD1+ on total CD8 cells | **-0.32 (0.03)** |  |  |  | 0.65 |
|  |  |  |  |  |  |
|  |  |  |  |  |  |
| Tim3+PD1+ on CD38-HLADR- CD8 cells | **-0.35 (0.02)** |  | **0.35** | **-0.79±0.32** | **0.02** |
|  |  |  |  |  |  |
|  |  |  |  |  |  |
| Tim3+PD1+ on CD38+HLADR- CD8 cells | **-0.30 (0.04)** |  |  |  | 0.55 |
|  |  |  |  |  |  |
|  |  |  |  |  |  |
| Tim3-PD1+ on CD38-HLADR+ CD8 cells | **-0.33 (0.03)** |  |  |  | 0.44 |
|  |  |  |  |  |  |
