# Supplementary material for: Expression of PD-1 and Tim-3 markers of T-cell exhaustion is associated with CD4 dynamics during the course of untreated and treated HIV infection
Source: PLoS One. 2018 Mar 8;13(3):e0193829. doi: 10.1371/journal.pone.0193829 (PMC5843247; doi:10.1371/journal.pone.0193829)
Supplement: S4 Table — (DOC) [file pone.0193829.s005.doc]

**S4 Table**. Bivariate analysis of associations between delta of immune parameters after LT (ΔLT) and delta CD4 during follow up in the absence of therapy.

|  |  | **Delta CD4** | | |
| --- | --- | --- | --- | --- |
|  |  |  |  |  |
| **ΔLT of** |  | Spearman Rho |  | p-value |
|  |  |  |  |  |
| **CD4 subsets** |  |  |  |  |
|  |  |  |  |  |
|  |  |  |  |  |
| CD31+CD45RA- cells |  | **-0.71** |  | 0.009 |
|  |  |  |  |  |
|  |  |  |  |  |
| CD31-CD45RA+ cells |  | **0.73** |  | 0.007 |
|  |  |  |  |  |
|  |  |  |  |  |
| CD95+ cells |  | **-0.69** |  | 0.007 |
|  |  |  |  |  |
|  |  |  |  |  |
| CD95+ (of CD31- cells) |  | **-0.82** |  | <0.0001 |
|  |  |  |  |  |
|  |  |  |  |  |
| CD95+ (of CD31-Ki67- cells) |  | **-0.78** |  | 0.001 |
|  |  |  |  |  |
|  |  |  |  |  |
| CD95+ (of CD31-Ki67+ cells) |  | **-0.66** |  | 0.01 |
|  |  |  |  |  |
| **CD8 subsets** |  |  |  |  |
|  |  |  |  |  |
|  |  |  |  |  |
| Tim3+PD1+ cells |  | **-0.77** |  | 0.002 |
|  |  |  |  |  |
|  |  |  |  |  |
| Tim3+PD1+ (of CD38+HLADR+ cells) |  | **-0.68** |  | 0.01 |
|  |  |  |  |  |
|  |  |  |  |  |
| Tim3+PD1+ (of CD38+HLADR- cells) |  | **-0.67** |  | 0.01 |
|  |  |  |  |  |
|  |  |  |  |  |
