# Supplementary material for: Expression of PD-1 and Tim-3 markers of T-cell exhaustion is associated with CD4 dynamics during the course of untreated and treated HIV infection
Source: PLoS One. 2018 Mar 8;13(3):e0193829. doi: 10.1371/journal.pone.0193829 (PMC5843247; doi:10.1371/journal.pone.0193829)
Supplement: S5 Table — (DOC) [file pone.0193829.s006.doc]

**S5 Table**. Bivariate and multivariate analysis of baseline levels of immune parameters associated with variation of CD4 count (ΔCD4) during follow up after initiation of cART.

|  | **Association with ΔCD4** | | | | |
| --- | --- | --- | --- | --- | --- |
|  |  | | | | |
|  | Bivariate analysis |  | Multivariate linear regression analysis | | |
|  |  |  |  |  |  |
| **Baseline values of** | **Pearson coefficient (p-value)** |  | **R of the model** | **Regression coefficient (ß±SE)** | **p-value** |
|  |  |  |  |  |  |
| CD38+HLADR- subset of CD8+ cells | **0.34 (0.02)** |  | **0.50** | **6.2±2.3** | **0.01** |
|  |  |  |  |  |  |
| Tim3+PD1+ on CD38-HLADR+ CD8+ cells | **0.34 (0.03)** |  |  |  | 0.30 |
|  |  |  |  |  |  |
| Tim3+ on CD45RA-CD31+ CD4+ cells | **0.30 (0.04)** |  |  |  | 0.27 |
|  |  |  |  |  |  |
