# Supplementary material for: Expression of PD-1 and Tim-3 markers of T-cell exhaustion is associated with CD4 dynamics during the course of untreated and treated HIV infection
Source: PLoS One. 2018 Mar 8;13(3):e0193829. doi: 10.1371/journal.pone.0193829 (PMC5843247; doi:10.1371/journal.pone.0193829)
Supplement: S6 Table — (DOC) [file pone.0193829.s007.doc]

**S6 Table**. Bivariate analysis of associations between delta of immune parameters after LT (ΔLT) and delta CD4 (ΔCD4) during follow up after initiation of cART.

|  |  | **ΔCD4** | | |
| --- | --- | --- | --- | --- |
|  |  |  |  |  |
| **ΔLT of** |  | Spearman Rho |  | p-value |
|  |  |  |  |  |
| **CD4 subsets** |  |  |  |  |
|  |  |  |  |  |
|  |  |  |  |  |
| Tim3+ on total CD4 cells |  | **-0.63** |  | 0.039 |
|  |  |  |  |  |
| Tim3+PD1- on CD31-CD45RA- subset |  | **-0.83** |  | 0.002 |
|  |  |  |  |  |
| Tim3+ on CD31-CD45RA+ subset |  | **-0.66** |  | 0.029 |
|  |  |  |  |  |
| PD1+ on CD31-CD45RA+ subset |  | **-0.59** |  | 0.060 |
|  |  |  |  |  |
| CD95+ on total CD4 cells |  | **-0.64** |  | 0.035 |
|  |  |  |  |  |
| **CD8 subsets** |  |  |  |  |
|  |  |  |  |  |
| PD1+ on total CD8 cells |  | **-0.61** |  | 0.036 |
|  |  |  |  |  |
| PD1+ on CD38-HLADR- subset |  | **-0.66** |  | 0.018 |
|  |  |  |  |  |
| PD1+ on CD38+HLADR- subset |  | **-0.58** |  | 0.048 |
|  |  |  |  |  |
