# Supplementary figures and images for: Expression of PD-1 and Tim-3 markers of T-cell exhaustion is associated with CD4 dynamics during the course of untreated and treated HIV infection
Source: PLoS One. 2018 Mar 8;13(3):e0193829. doi: 10.1371/journal.pone.0193829 (PMC5843247; doi:10.1371/journal.pone.0193829)

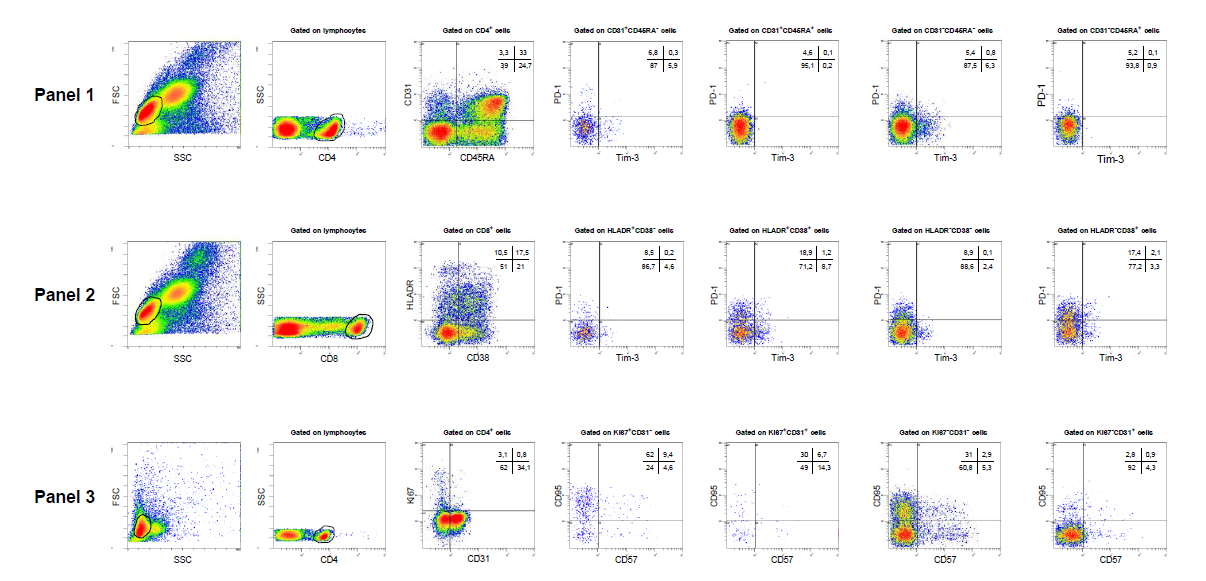

Supplement: S1 Fig — Numbers inside the dot-plots represents percentages of cells in each quadrant of the plot. (TIF) [file pone.0193829.s008.tif]
