# Supplementary material for: Expression of PD-1 and Tim-3 markers of T-cell exhaustion is associated with CD4 dynamics during the course of untreated and treated HIV infection
Source: PLoS One. 2018 Mar 8;13(3):e0193829. doi: 10.1371/journal.pone.0193829 (PMC5843247; doi:10.1371/journal.pone.0193829)
Supplement: S2 Fig — Upper graphs show levels of different CD4 subsets on the basis of CD45RA, CD31 and Ki67 expression. Middle graph shows levels of PD1 and Tim3 markers on different subsets of CD4 cells defined by CD31 and CD45RA markers; and lower graph the levels of activation/apoptosis (CD95) and senescence (CD57) on different subsets of CD4 cells defined by CD31 and Ki67 markers. Statistically significant differences between the three groups (by Kruskall-Wallis test) are marked by an asterisk, significant differences between cART naïve and cART groups of patients (by Mann-Whitney U test) are marked by ¶ symbol, and significant differences of each patient´s groups with respect to healthy controls (by Mann-Whitney U test) are marked by # symbol. (PPT) [file pone.0193829.s009.ppt]

## Slide 1
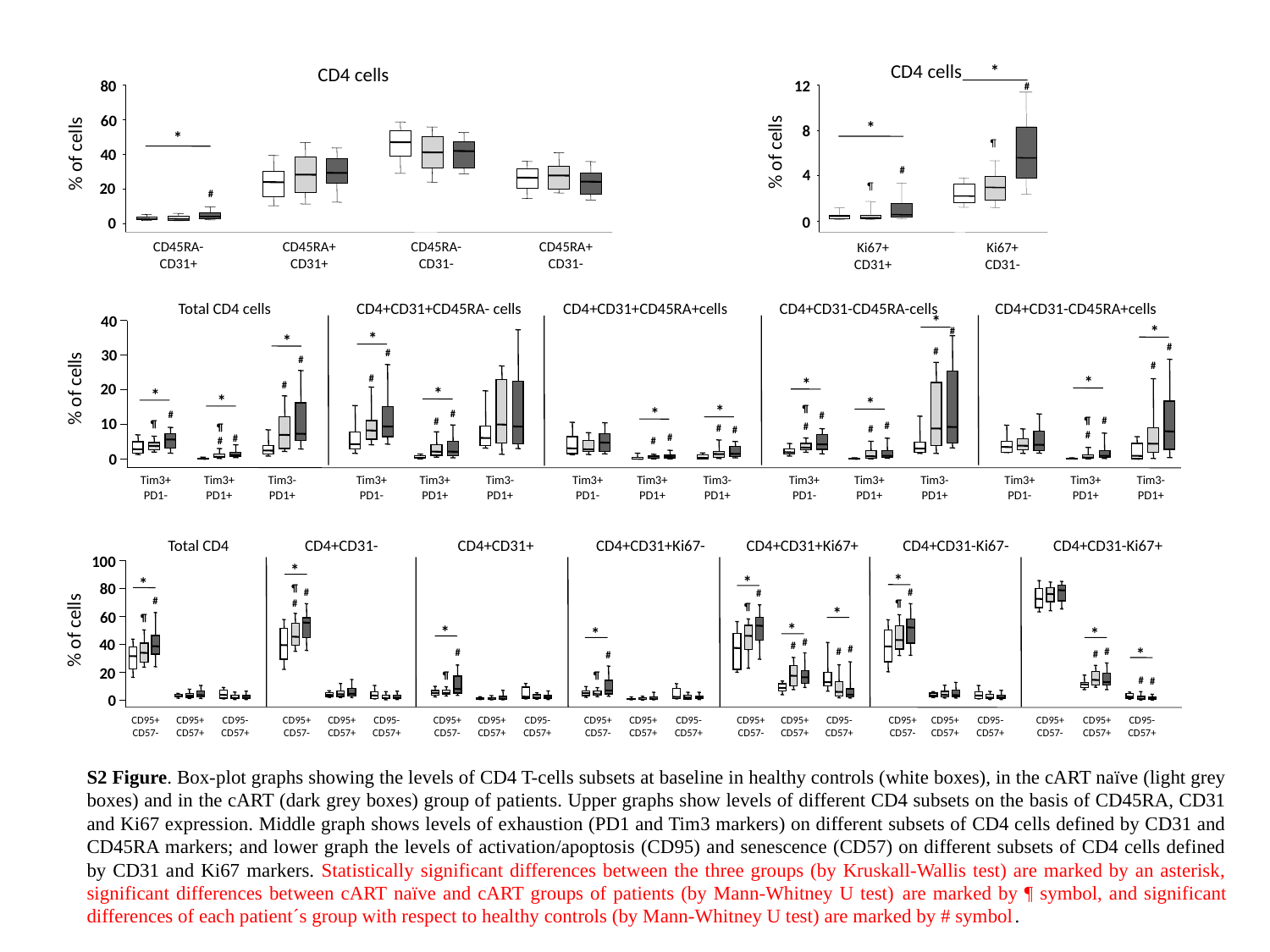

CD4 cells
*
CD4 cells
80
12
#
60
*
8
*
¶
% of cells
% of cells
40
#
4
20
¶
#
0
0
CD45RA-
CD31-
CD45RA-
CD31+
CD45RA+
CD31+
CD45RA+
CD31-
Ki67+
CD31+
Ki67+
CD31-
Total CD4 cells
CD4+CD31+CD45RA- cells
CD4+CD31+CD45RA+cells
CD4+CD31-CD45RA-cells
CD4+CD31-CD45RA+cells
*
40
*
#
*
*
#
#
30
#
#
#
#
*
*
% of cells
#
20
*
*
*
*
*
*
¶
#
#
#
10
¶
#
#
¶
#
#
¶
#
#
#
#
#
#
#
#
0
Tim3+
PD1-
Tim3+
PD1+
Tim3-
PD1+
Tim3+
PD1-
Tim3+
PD1+
Tim3-
PD1+
Tim3+
PD1-
Tim3+
PD1+
Tim3-
PD1+
Tim3+
PD1-
Tim3+
PD1+
Tim3-
PD1+
Tim3+
PD1-
Tim3+
PD1+
Tim3-
PD1+
CD4+CD31+
Total CD4
CD4+CD31-
CD4+CD31+Ki67-
CD4+CD31+Ki67+
CD4+CD31-Ki67-
CD4+CD31-Ki67+
100
*
*
*
*
80
¶
#
#
#
#
#
¶
¶
*
60
¶
*
% of cells
*
*
*
40
#
#
#
*
#
#
#
#
#
20
¶
¶
#
#
0
CD95+
CD57-
CD95+
CD57+
CD95-
CD57+
CD95+
CD57-
CD95+
CD57+
CD95-
CD57+
CD95+
CD57-
CD95+
CD57+
CD95-
CD57+
CD95+
CD57-
CD95+
CD57+
CD95-
CD57+
CD95+
CD57-
CD95+
CD57+
CD95-
CD57+
CD95+
CD57-
CD95+
CD57+
CD95-
CD57+
CD95+
CD57-
CD95+
CD57+
CD95-
CD57+
S2 Figure. Box-plot graphs showing the levels of CD4 T-cells subsets at baseline in healthy controls (white boxes), in the cART naïve (light grey boxes) and in the cART (dark grey boxes) group of patients. Upper graphs show levels of different CD4 subsets on the basis of CD45RA, CD31 and Ki67 expression. Middle graph shows levels of exhaustion (PD1 and Tim3 markers) on different subsets of CD4 cells defined by CD31 and CD45RA markers; and lower graph the levels of activation/apoptosis (CD95) and senescence (CD57) on different subsets of CD4 cells defined by CD31 and Ki67 markers. Statistically significant differences between the three groups (by Kruskall-Wallis test) are marked by an asterisk, significant differences between cART naïve and cART groups of patients (by Mann-Whitney U test) are marked by ¶ symbol, and significant differences of each patient´s group with respect to healthy controls (by Mann-Whitney U test) are marked by # symbol.
