# Supplementary material for: Expression of PD-1 and Tim-3 markers of T-cell exhaustion is associated with CD4 dynamics during the course of untreated and treated HIV infection
Source: PLoS One. 2018 Mar 8;13(3):e0193829. doi: 10.1371/journal.pone.0193829 (PMC5843247; doi:10.1371/journal.pone.0193829)
Supplement: S3 Fig — Upper graph shows levels of different CD8 subsets on the basis of expression of activation markers CD38 and HLADR. Lower graph shows levels of PD1 and Tim3 markers on different subsets of CD8 cells defined by the expression of activation markers CD38 and HLA DR. Statistically significant differences between the three groups (by Kruskall-Wallis test) are marked by an asterisk, significant differences between cART naïve and cART groups of patients (by Mann-Whitney U test) are marked by ¶ symbol, and significant differences of each patient´s groups with respect to healthy controls (by Mann-Whitney U test) are marked by # symbol. (PPT) [file pone.0193829.s010.ppt]

## Slide 1
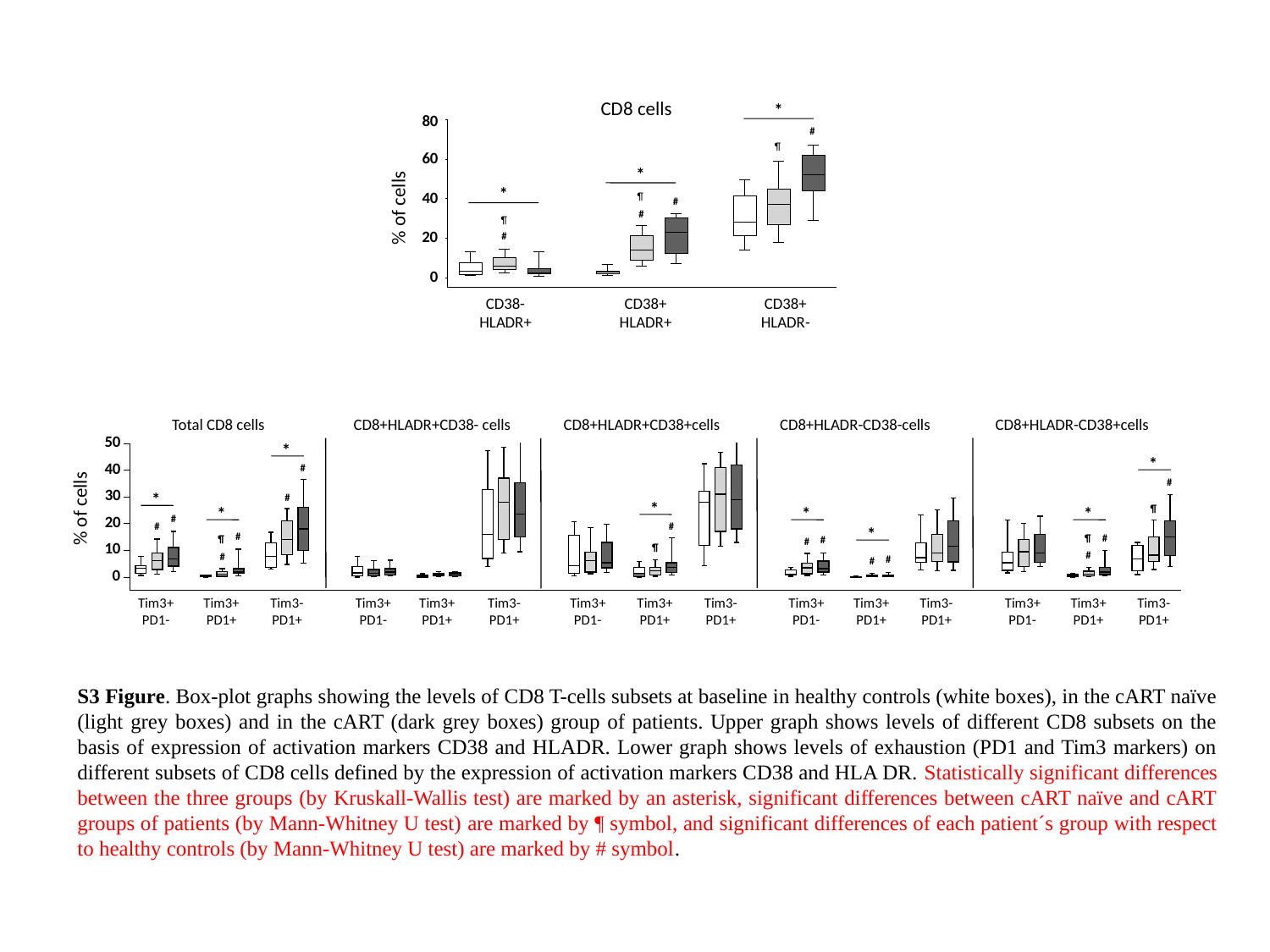

CD8 cells
*
80
#
¶
60
*
*
40
¶
#
% of cells
#
¶
20
#
0
CD38-
HLADR+
CD38+
HLADR+
CD38+
HLADR-
Total CD8 cells
CD8+HLADR+CD38- cells
CD8+HLADR+CD38+cells
CD8+HLADR-CD38-cells
CD8+HLADR-CD38+cells
50
*
*
40
#
#
30
*
#
% of cells
*
¶
*
*
*
20
#
#
#
*
#
#
¶
¶
#
#
10
¶
#
#
#
#
0
Tim3+
PD1-
Tim3+
PD1+
Tim3-
PD1+
Tim3+
PD1-
Tim3+
PD1+
Tim3-
PD1+
Tim3+
PD1-
Tim3+
PD1+
Tim3-
PD1+
Tim3+
PD1-
Tim3+
PD1+
Tim3-
PD1+
Tim3+
PD1-
Tim3+
PD1+
Tim3-
PD1+
S3 Figure. Box-plot graphs showing the levels of CD8 T-cells subsets at baseline in healthy controls (white boxes), in the cART naïve (light grey boxes) and in the cART (dark grey boxes) group of patients. Upper graph shows levels of different CD8 subsets on the basis of expression of activation markers CD38 and HLADR. Lower graph shows levels of exhaustion (PD1 and Tim3 markers) on different subsets of CD8 cells defined by the expression of activation markers CD38 and HLA DR. Statistically significant differences between the three groups (by Kruskall-Wallis test) are marked by an asterisk, significant differences between cART naïve and cART groups of patients (by Mann-Whitney U test) are marked by ¶ symbol, and significant differences of each patient´s group with respect to healthy controls (by Mann-Whitney U test) are marked by # symbol.
