# Supplementary material for: Expression of PD-1 and Tim-3 markers of T-cell exhaustion is associated with CD4 dynamics during the course of untreated and treated HIV infection
Source: PLoS One. 2018 Mar 8;13(3):e0193829. doi: 10.1371/journal.pone.0193829 (PMC5843247; doi:10.1371/journal.pone.0193829)
Supplement: S4 Fig — Pearson r or Spearman Rho (σ) coefficients and p-value are shown in each scatter plot. (PPT) [file pone.0193829.s011.ppt]

## Slide 1
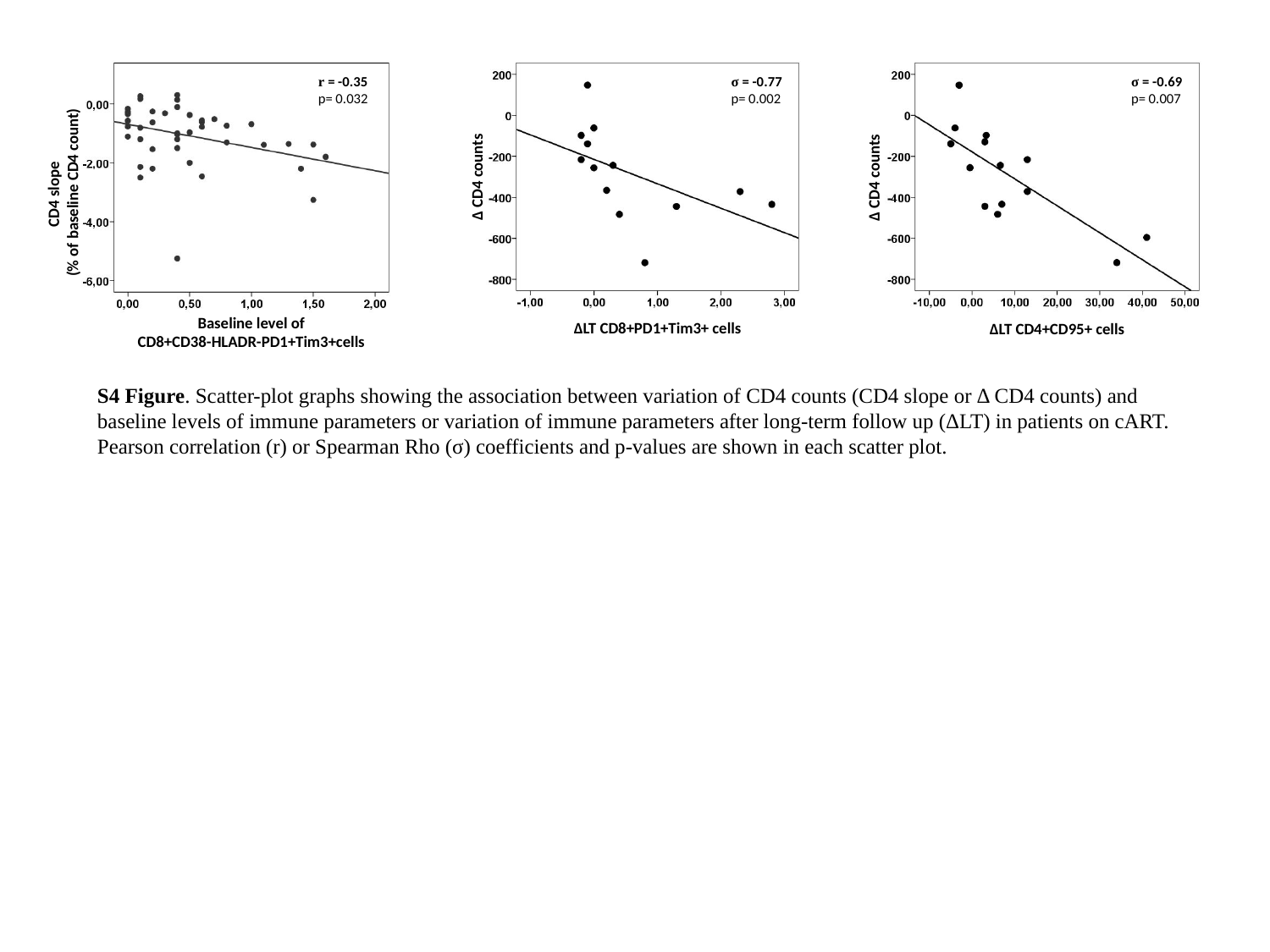

CD4 slope
(% of baseline CD4 count)
Baseline level of
CD8+CD38-HLADR-PD1+Tim3+cells
Δ CD4 counts
ΔLT CD8+PD1+Tim3+ cells
Δ CD4 counts
ΔLT CD4+CD95+ cells
r = -0.35
p= 0.032
σ = -0.77
p= 0.002
σ = -0.69
p= 0.007
S4 Figure. Scatter-plot graphs showing the association between variation of CD4 counts (CD4 slope or Δ CD4 counts) and baseline levels of immune parameters or variation of immune parameters after long-term follow up (ΔLT) in patients on cART. Pearson correlation (r) or Spearman Rho (σ) coefficients and p-values are shown in each scatter plot.
